# In Vivo Evaluation of Wound Healing and Anti-Inflammatory Activity of 80% Methanol Crude Flower Extract of *Hagenia abyssinica* (Bruce) J.F. Gmel in Mice

**DOI:** 10.1155/2020/9645792

**Published:** 2020-03-30

**Authors:** Teshome Fentik Belachew, Seyfe Asrade, Mestayt Geta, Engidaw Fentahun

**Affiliations:** ^1^Department of Pharmacy, Debre Birhan Health Science College, Debre Birhan, Ethiopia; ^2^Department of Pharmacology, School of Pharmacy, University of Gondar, Gondar, Ethiopia; ^3^Department of Human Anatomy, School of Medicine, University of Gondar, Gondar, Ethiopia

## Abstract

**Background:**

*Hagenia abyssinica* (Bruce) J.F. Gmel (Rosaceae) is distributed in the highlands of Ethiopia. The flowers of *Hagenia abyssinica* (Bruce) J.F. Gmel are used traditionally to treat wound. However, there was no scientific report on wound healing activity of *Hagenia abyssinica* (Bruce) J.F. Gmel. Thus, this study was initiated to investigate the wound healing and anti-inflammatory activities of 80% methanol crude extract of flowers of *Hagenia abyssinica* in mice.

**Objective:**

The objective of this study was to evaluate the wound healing and anti-inflammatory activity of 80% methanol crude flower extract of *Hagenia abyssinica* in mice.

**Methods:**

Air-dried flowers of *Hagenia abyssinica* were grounded and macerated three times successively by 80% methanol. The dried extract was fractionated with chloroform, ethyl acetate, and water. Phytochemical screening tests were performed according to established procedures. The crude extract and solvent fractions were formulated as ointments. Wound healing activity of the crude extract was evaluated using excision and incision wound models, and the wound healing activities of solvent fractions were evaluated by using the excision wound model. The anti-inflammatory activity of the 80% methanol extract of *Hagenia abyssinica* was evaluated using carrageenan-induced hind paw edema model in mice.

**Result:**

The 2000 mg/kg test dose of the 10% (w/w) crude extract ointment was safe in rats. Both the 5% (w/w) and 10% (w/w) crude extract ointment-treated groups showed significant wound contraction starting from the day 4^th^. Both 5% (w/w) and 10% (w/w) crude extract ointments showed significant (*P* < 0.001) increment of tensile strength compared to the negative control. The 10% (w/w) aqueous and ethyl acetate fraction ointment revealed high (*P* < 0.001) percentage of wound contraction. The 100 mg/kg, 200 mg/kg, and 400 mg/kg oral administration of the crude extract had significant inhibition of the paw edema in mice of carrageenan-induced inflammation.

**Conclusion:**

The results of this study evidenced that both 5% w/w and 10% w/w 80% methanol extract ointment of the flowers of *Hagenia abyssinica* have wound healing and anti-inflammatory effects.

## 1. Background

Wound is defined as the cellular and anatomic disruption of structure and function of tissue. It ranges from a simple break in the epithelial integrity of the skin, or it can be deeper, extending into subcutaneous tissue with damage to other structures such as tendons, muscles, vessels, nerves, parenchymal organs, and bone. It is caused by chemical, physical, microbial, thermal, or immunological damage of tissues [1, 2].

Wound can be classified as acute and chronic. Acute wounds represent the injured skin (e.g., resulted from burns and chemical injuries) that heals through the regular phases of wound repair; in contrast, chronic wounds need a longer healing time. The time course of healing usually ranges from 5 to 10 days [2, 3]. Chronic wounds fail to progress through the normal stages of healing, and they cannot be repaired in an orderly and timely manner. The healing process is incomplete and disturbed by various factors, which prolong to one or more stages [4].

The wound healing is a normal biological process, and it involves four complex steps: homeostasis/coagulation; inflammation, migration, and proliferation; reepithelialization; and restoration. Each phase of the wound healing process is influenced by a series of mediators such as platelets and cytokines, inflammatory cells, cellular and extracellular matrix, proteinases, growth factors, and inhibitors [5]. The hemostatic and inflammatory stages take place immediately after damage, but the inflammatory stage may last for up to 6 days. The proliferation stage is considered as the beginning of angiogenesis and the development of the extracellular matrix. A prolonged time of the inflammatory and/or proliferative phase will result in a hindered healing, encouraging excessive scar tissue establishment. The remodeling stage typically initiates 3 weeks after damage. Remodeling consists of the deposition of the matrix and its subsequent changes over time. It occurs throughout the entire wound repair process as fibrin clot formed in the early inflammatory phase is replaced by the granulation tissue that is rich in type III collagen and blood vessels during the proliferative phase and subsequently replaced by a collagenous scar predominantly of type I collagen with much less mature blood vessels [6, 7].

*Hagenia abyssinica* (Bruce) J.F. Gmel is a slender tree, 5 to 25 m tall, with a short trunk and thick branches; with branchlets covered in silky brown hairs and ringed with leaf scars. Its bark is thick, brown or reddish-brown, and readily peels. It has compound leaves with 3–6 pairs of leaflets plus a terminal leaflet. The flowers are greenish, or white, turning reddish with maturity, and they form handsome multibranched terminal. It is found in the Democratic Republic of Congo, Sudan, Ethiopia, Malawi, Zambia, Zimbabwe, Kenya, Tanzania, Uganda, Burundi, and Rwanda [8, 9].

The flower and leaf powders are applied with honey on the wound. The leaves, root, and bark decoction with cold water is drunk to treat stomachache, typhoid, diarrhea, and cough. The bark powders and root are macerated and drunk for treatment of cough, fever, bronchitis, throat disease, cancer, and malaria. The powder of barks with other plants is applied on skin for dermatological disease. A flower infusion or decoction is drunk for treatment of intestinal worms, epilepsy, evil eye, hepatitis, and sexually transmitted disease. The infused flowers are applied for the healing of injured skin. The juice of flower and leaves is used for hypertension and diabetes mellitus [8, 9].

According to the Ethnobotanical study, leaves and flowers of *Hagenia abyssinica* have been used traditionally for the treatment of wound in Abaya district, Borana Zone, Oromia Regional State, Hawassa city, Regional state of Southern Nation and nationalities, Kofele and Bale district Oromia Regional State, and Debark district North Gondar Zone of the Amhara Region [8–10]. However, the plant has not been explored scientifically for its wound healing activity. Hence, this study was designed to investigate the wound healing activity of crude extract and their fractions of *Hagenia abyssinica* (Bruce) J.F. Gmel flowers by using different wound models.

## 2. Methods

### 2.1. Collection of Plant Materials

The flowers of *H. abyssinica* were collected from Kosoye located in Amhara Region, north west Ethiopia, and 15 km from Gondar town in February, 2019. The plant was identified and authenticated by Botanist at Department of Biology, College of Natural and Computational science, University of Gondar, for future reference with a voucher specimen number 001TF/2019.

### 2.2. Experimental Animals

A total of 132 healthy, adult swiss albino mice (either sex of 25–35 g, 8–10 weeks of age) and 10 adult healthy, female Wistar rats (180–200 gm, 3–4-month of age) were procured from the animal house of the Ethiopian Public Health Institute. The animals were kept in cages and housed in a standard animal house under natural 12/12 h light dark cycle at room temperature and provided with pellet diet and water ad libitum in the animal house of Department of Pharmacology, University of Gondar. All mice and rats were allowed to acclimatize the laboratory condition for a week before the starting of the experiment. Animal handling and care was carried out throughout the experiment following international laboratory animal use and care guidelines. At the end of the experiment, the animals were sacrificed by high dose of halothane [11].

### 2.3. Preparation of the Crude Extract

The *H. abyssinica* (Bruce) J.F. Gmel flowers were washed under running tap water to remove the surface pollutants and shade-dried at room temperature. The dried flowers were coarsely powdered in a grinder, and 1000 g of the powder was macerated with 8000 ml of 80% methanol for three days in a conical flask with occasional stirring (every 24 hours) and shaking (120 rpm) [12, 13]. Then after, the extract was separated from the marc by using muslin cloth and further filtered by Whatman filter paper No. 1. The residue further macerated with the methanol for three more days to exhaustively extract the plant material and filtered. The collective filtrates were placed in a drying oven at 40°C until dry. The filtrate was frozen overnight using deep freezer, and then freeze was dried in a lyophilizer. It was stored in screw cap vials in a refrigerator at −4°C until used for formulation of ointments and solvent fractionation [14, 15].

#### 2.3.1. Solvent Fraction of the Crude Extract

Two hundred forty grams of crude extract of the flowers of *H. abyssinica* (Bruce) J.F. Gmel was suspended in 1200 ml of distilled water and slightly shaken to mix completely with solvent. The mixture was transferred in to a separator funnel. Then, equal volume of chloroform was added to it. The new mixture was shaken gently to mix and allowed to settle for some times until it forms two layers, and then the chloroform fraction was collected; the procedure was repeated twice as described above. Then, aqueous residue was further fractionated three times with ethylacetate to obtain ethylacetate fraction. The upper layer was ethylacetate, which was separated from aqueous portion. Finally, the aqueous solution was collected as the third fraction. The ethylacetate and chloroform fraction residue was dried by dry oven at 40°C. The aqueous fraction was frozen in a refrigerator overnight and then dried using a lyophilizer. All fractions were stored in screw cap vials in a refrigerator at −4°C until being used for formulation of ointments [16, 17].

### 2.4. Ointment Formulation

5% w/w and 10% (w/w) strength of crude extract and chloroform, ethylacetate and aqueous fractions, and simple ointment were prepared by the fusion method based on British Pharmacopoeia [18] and are shown in Table 1.

The 200 g of simple ointment base was prepared by placing hard paraffin (10 g) in a beaker and melted over water bath. The other ingredients such as cetostearyl alcohol (10 g), white soft paraffin (170 g), and wool fat (10 g) were added in the descending order of melting point, respectively, after removing from melting. All the ingredients were melted over a water bath with constant stirring until they became homogeneous. The mixture was removed from the heat and stirred until cold. To prepare hydroalcoholic extract ointment, 10 g and 20 g of the powdered extracts were incorporated into 190 g and 180 g of simple ointment base portion by portion to prepare 5% and 10% (w/w) ointment, respectively, by levigation on the surface of the ointment slab to make ointment of uniform consistency and smooth texture. Finally, the extract ointment was transferred to a clean container for topical application during the experiment. The control ointment, 100 gm of the entire base ingredients, was taken and treated in the same way to formulate ointment without an active ingredient [13, 18]. The same procedure was used for each fraction ointment preparation.

### 2.5. Preliminary Phytochemical Screening

Standard screening test of the extract was carried out for various plant constituents. The crude extract was screened for the presence or absence of secondary metabolites such as reducing sugars, alkaloids, steroidal compounds, phenolic compounds, cardiac glycosides, flavonoids, saponins, tannins, and anthraquinones using standard procedures.

#### 2.5.1. Test for Alkaloids

The extract (0.2 g) was dissolved in dilute hydrochloric acid. The solution was filtered, and a few drops of Dragendorff's reagent were added. The treated solutions were observed for any precipitation. Similarly, to a portion of filtered solution, a few drops of Mayer's reagent were added. The treated solutions were observed for any precipitation.

#### 2.5.2. Test for Flavonoids

Five ml of ethyl acetate was added to a solution of 0.5 g of the extract in water. The mixture was shaken, allowed to settle, and inspected for the production of yellow color in the organic layer, which is taken as positive for free flavonoids.

#### 2.5.3. Test for Saponins

The hydroalcoholic extract (0.5 g) was dissolved in 10 ml of distilled water in a test tube. The test tube was corked and shaken vigorously for about 30 sec. The test tube was allowed to stand vertically and observed over a 30 min period of time. If froth above the surface persists after 30 min, the sample is suspected to contain saponins.

#### 2.5.4. Test for Tannins

A portion of the alcoholic extract was dissolved in water. The solution was clarified by filtration. 10% ferric chloride solution was added to the clear filtrate. This was observed for a change in color to bluish black.

#### 2.5.5. Test for Glycosides

0.5 g of crude extracts was dissolved separately in 5 mL of methanol. 10 mL of 50% HCl was added to 2 mL of each extract in test tubes. The mixtures were heated in a boiling water bath for 30 min. 5 mL of Fehling's solution was added, and the mixtures were boiled for 5 min to give a brick red precipitate as an indication for the presence of glycosides.

#### 2.5.6. Test for Phenols

0.5 g of each crude extracts was put in a different test tube and treated with a few drops of 2% of FeCl_3_; bluish green or black coloration indicated the presence of phenols.

#### 2.5.7. Test for Steroids

0.5 g of each crude extracts was dissolved in 5 mL of methanol. 1 mL of the extract was treated with 0.5 mL of acetic acid anhydride and cooled in ice. This mixed with 0.5 mL of chloroform and 1 mL of concentrated sulphuric acid was then added carefully by means of a pipette. At the separation level of the two liquids, reddish-brown rings were formed, as indication of the presence of steroids.

#### 2.5.8. Test for Terpenoids

0.5 g of each crude powders was separately dissolved in 5 mL of methanol. 2 mL of the extract was treated with 1 mL of 2,4-dinitrophenyl hydrazine and was dissolved in 100 mL of 2M HCl. Yellow-orange colorations were observed as an indication of terpenoids.

#### 2.5.9. Test for Anthraquinones

The extract of the plant material (equivalent to 100 mg) was shaken vigorously with 10 ml of benzene and filtered, and 5 ml of 10% ammonia solution was added to the filtrate. The mixture was shaken and observed for the presence of a pink, red, or violet color in the ammonia (lower) phase that indicates the presence of free anthraquinones.

### 2.6. Acute Dermal Toxicity

Acute oral toxicity test done in the previous study did not show any signs and symptoms of toxicity and death of animals in a single-dose administration of 2000 mg/kg [19].

Acute dermal toxicity test was performed according to the OECD draft guideline number 434. A total of ten female Wistar rats aged between 8 and 12 weeks were used. They were divided into two groups of five animals each for treatment and control groups. Animals with normal skin texture were housed in a cage and acclimatized to the laboratory condition for one week prior to the test. Following acclimation, around 10% of the body surface area fur was shaved 24 h before the study from the dorsal area of the trunk of the test animals. First, a sighting study was performed to determine the starting dose by applying 2000 mg/kg of the 10% extract ointment. There was no death or skin irritation within 24 h, and then four additional rats from each group was used; the same dose of the extract ointment was applied. At the end of the exposure period (24 hours), residual test substance was removed and the animals were observed for development of any adverse skin reactions daily for 14 days [20, 21].

### 2.7. Grouping and Dosing of Experimental Animals

Four groups of mice containing six in each were used for the excision model. Animals in group I were treated topically with nitrofurazone (0.2%) ointment. Groups II and III were received 10% w/w and 5% w/w methanol extract ointments, respectively, and the animals in group IV were treated with simple ointment. Five groups of mice containing six in each were used for the incision wound model. The animals of groups I–IV were treated in a similar fashion with the excision wound model, but animals in group V were left untreated (served as untreated negative control).

For evaluation of solvent fractions wound healing activity, eight groups of mice (each with six) and circular excision wound model were used. Group I was treated with simple ointment (served as a negative control); groups II and III were treated with 5 w/w% and 10% w/w of aqueous ointment, respectively; groups IV and V were treated with 5% w/w and 10% w/w of ethylacetate ointment, respectively; groups VI and VII were treated with 5% w/w and 10% w/w of chloroform ointment, respectively; group VIII was treated with 0.2% w/w nitrofurazone (served as a positive control) [22].

For evaluation of anti-inflammatory activity, there were five groups of six mice each. Group I is normal saline solution group, group II received 100 mg/kg body weight dose of the extract solution in saline, group III received 200 mg/kg body weight dose of the extract solution in saline, group IV received 400 mg/kg body weight dose of the extract solution in saline, and group V received 5 mg/kg body weight dose of indomethacin in saline solution [11, 23].

### 2.8. Excision Wound Model

**Figure 1:**
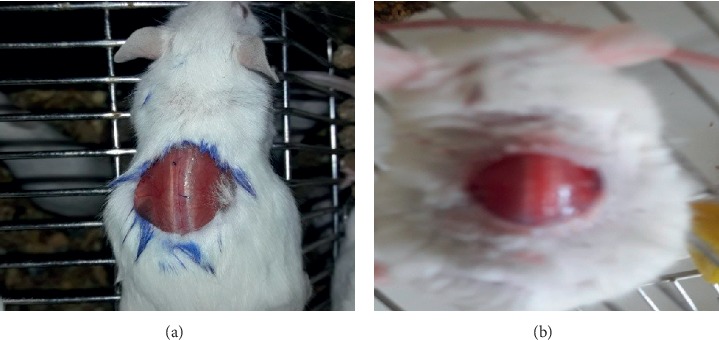
Excision wound on day 0.

The mice were anesthetized with intraperitoneal 50 mg/kg ketamine and 5 mg/kg diazepam [24]. Before wound area preparation, the dorsal furs of the animals were shaved with shaving machine. Then, their fur from the dorsothoracic area was removed. A 314 mm^2^ circular mark was prepared using a permanent marker. Then, full thickness of this circular mark was excised using forceps and scissors to form wound (Figure 1). This was considered as day 0. Starting from day one, the mice were treated as described in the grouping and dosing. For the evaluation of wound healing activity of the chloroform, ethyl acetate, and aqueous fractions, the same wound area was created and ointments were applied as described in the grouping and dosing. All the preparations were applied daily to the wound area until the wound in the test groups completely healed. Wound area was measured every 2 days using transparent sheet and permanent marker. The transparent sheet was placed on 1 mm^2^ scale graph paper and traced out. The wound healing activities of crude extract and solvent fractions were assessed by the period of epithelialization and percentage of wound contraction [25–27]. The percentage of wound contraction calculated for crude extract and solvent fractionation is as follows:

(1) $$\begin{array}{c} \%\text{wound contraction}=\frac{\text{wound area on day }0\;-\text{ wound area on day}\;n}{\text{wound area on day }0}\times 100, \end{array}$$

where *n* = the days when measurement was taken.

#### 2.8.1. Epithelialization Period Measurement

Complete epithelization period was calculated as the number of days required for falling off of the dead tissue remnants without any residual raw wound [28, 29].

### 2.9. Linear Incision Wound Model

**Figure 2:**
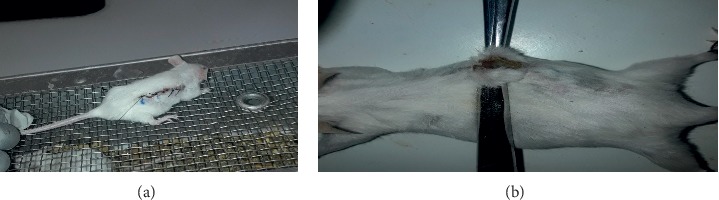
Incision wound creation (a) and tensile strength measurement (b) during the experiment.

Thirty mice were anesthetized, and their fur was shaved similarly to that in the excision wound model. Three cm long, linear-par vertebral incision was made through the full thickness of the skin on either side of the vertebral column at a distance of 1 cm from the midline. The skin was kept together and stitched using black braided silk (no. 00) and a curved needle (no. 11) at the intervals of 1 cm [24, 30]. This wounding day was considered day 0 (Figure 2(a)). Starting from day one, the ointments were applied as indicated in the grouping and dosing. The treatments were applied topically once per day for 9 days. The suture was removed on the 8^th^ postwounding day, and the tensile strength of the skin was measured on the 10^th^ day to measure the extent of healing. It was measured through continuous water flow technique by considering the gram of water required to break the skin (Figure 2(b)). Tensile strength was calculated using the following formula [29, 31]:

(2) $$\begin{array}{c} \frac{\text{TS extract}}{\text{standard}}\;=\;\frac{\text{TS extract}/\text{standard}-\text{TSso}}{\text{TSso}}\times 100,\\ \text{TSso }=\;\frac{\text{TSso}-\text{TSl}.\text{u}}{\text{TSl}.\text{u}}\times 100, \end{array}$$

where TS = tensile strength, so = simple ointment, and l.u = left untreated.

### 2.10. Evaluation of In Vivo Anti-Inflammatory Activity

#### 2.10.1. Carrageenan-Induced Paw Edema Model

Anti-inflammatory activity of the crude extract was determined using carrageenan-induced paw edema in mice according to the standard method [32]. The mice were fasting overnight with free access to water, and the basal volume of the right hind paw of each mouse was determined before oral administration of any drug using plethysmometer [33].Then, the treatments were given as indicated in the grouping and dosing. Edema was induced by subplantar injection of 0.05 mL of 1% freshly prepared solution of carrageenan in normal saline into the left hind paws of each mouse of all the groups. The change in volume of the paw was measured after 1, 2, 3, and 4 hours of the administration of carrageenan injection by using plethysmometer. An increase in paw volume at 1, 2, 3, and 4 h after carrageenan injection was considered as the parameter for measurement of inflammation. The average foot swelling in extract treated mice as well as standard was compared with that of the negative control, and the percent inhibition (anti-inflammatory activity) of edema was determined using the following formula [29, 34, 35]:

(3) $$\begin{array}{c} \text{percentage inhibition of edema }=\;\frac{\text{Co}-\text{Ct}}{\text{Co}}\times 100, \end{array}$$

where *C*o is the average inflammation (hind paw edema) of the control group at a given time and *C*t is the average inflammation of the plant extract or indomethacin-related mice at the same time [34, 36].

### 2.11. Statistical Analysis

All the results were expressed as mean ± SEM for each group. All the grouped data were statistically evaluated, and the significance of various treatments was calculated using one-way ANOVA followed by Tukey's HSD post hoc test. The results were considered statistically significant at 95% confidence level and *P* value < 0.05. All data processing was done using SPSS data analysis software version 24.

## 3. Result

Eighty percent methanol crude extract of the plant flowers resulted in 18.67% yields of crude extract, and 240 gm of crude extract fractionate produces 47.91%, 16.67%, and 33.33% aqueous, chloroform, and ethyl acetate fraction, respectively.

### 3.1. Phytochemical Constituents of the Crude Extract

According to the qualitative phytochemical screening study, the crude extract of the flower of *Hagenia abyssinica* is found to be positive for the presence of secondary metabolite, as shown in Table 2.

### 3.2. Acute Dermal Toxicity

In the acute dermal toxicity test, application of 10% (w/w) crude extracts ointment (CEO) with a limit dose of 2000 mg/kg was found to be safe. After 24 hours of application in the shaved area, there was no sign of erythema and edema. Neither mortality nor sign of toxicity was observed in rats when monitoring for 14 days after application of 10% CEO.

### 3.3. Evaluation of Wound Healing Activity

#### 3.3.1. Excision Wound Model

Topical application of ointments of the 80% methanol crude extracts of *H. abyssinica* flowers showed a significant effect in the wound healing process compared to the negative control (NC) in mice. The progressive wound contraction induced by treatment with 5% and 10% (w/w) CEO, NC, and nitrofurazone 0.2% ointment (NF) is shown in Table 3. The CEO facilitates wound contraction significantly at both dose levels from the 4^th^ day to 14^th^ day as compared to NC. The 5% (w/w) CEO-treated group showed significant (*P* < 0.01) effect of wound contraction on days 4^th^ and 6^th^ compared to the negative control.

The 10% (w/w) CEO-treated group showed significant (*P* < 0.001) wound contraction starting from day 4^th^. There was no significant wound contraction difference among groups treated by 5% (w/w) CEO, 10% (w/w) CEO, and NF but significant compared to NC.

**Figure 3:**
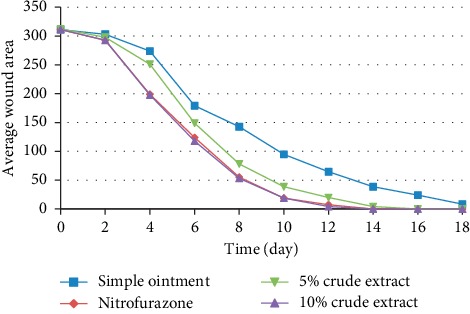
Effect of 80% methanolic crude extract of *H. abyssinica* flowers on the average wound area of excision wound model in mice.

**Figure 4:**
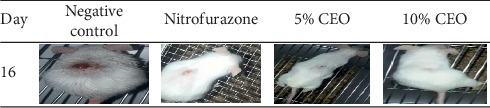
Excision wound on different days. CEO is 80% methanol extracts of flower of *H. abyssinica*.

The percentage of wound closure was higher in groups treated by 10% (w/w) CEO compared to the 5% (w/w) CEO on all postwounding days but not significant. The 5% (w/w) and 10% (w/w) CEO- and NF-treated groups showed very much close percentage of wound closure on the 14^th^ day postwounding period which was 98.67%, 100%, and 100%, respectively (Figure 3). The complete wound closure was observed in 10% (w/w) CEO- and NF-treated groups within 14 days (Figure 4).

In the excision wound model, groups treated by the 10% aqueous and ethyl acetate fraction ointment showed significant (*P* < 0.001) wound contraction from day 4^th^ onwards. The group treated with 10% (w/w) AQFO had a greater wound contraction rate compared to the positive control in days 6^th^, 8^th^, and 10^th^ but failed to reach statistical significance.

There was significant difference between 5% (w/w) AQFO and 10% EAFO starting from day 4^th^ on wards, but there was no significant difference between the low dose of AQFO and EAFO.

Mice treated with 5% (w/w) and 10% (w/w) CHFO showed significant (*P* < 0.05 and *P* < 0.01, respectively) wound contraction starting from days 8^th^ and 4^th^, respectively, compared to the NC (simple ointment). 10% (w/w) EAFO showed significant (*P* < 0.001) wound contraction difference compared to the 10% (w/w) CHFO starting from day 4 to day 8. There was comparable reduction in wound area among groups treated by NF, 10% (w/w) EAFO, and 10% (w/w) AQFO from day 4^th^ onwards. The highest rate of wound closure observed in a group treated by 10% (w/w) EAFO started from the day 4^th^ to 12^th^ compared to the other groups. The result of solvent fraction is as shown in Table 4.

*(1) Epithelization Period*. The time of complete epithelialization was short in 5% (w/w) and 10% (w/w) CEO-treated groups as compared to negative control group (simple ointment), as shown Table 5. The mean period of epithelialization was 18.67, 13.00, 14.67, and 12.67 days for the control group, NF, 5% (w/w), and 10% (w/w) CEO, respectively. Groups treated with 5% (w/w) and 10% (w/w) CEO and NF showed 21.42%, 32.14%, and 30.37% decrease in the epithelialization period.

Groups treated with 5% and 10 w/w ointment of each fraction had short period of epithelization compared to NC. The period of epithelization of groups treated with 10% EAFO was statistically significant (*P* < 0.001) compared to the NC. Mice treated by 5% EAFO, 10% AQFO, and NF also showed significant (*P* < 0.01) reduction of the epithelization period compared to negative control. The period of epithelization was insignificant among groups treated by each of 5% (w/w) and 10 (w/w) fraction ointments. Group treated with 10% EAFO had shortest period of epithelization (14 days) and highest percentage of decrease in epithelization period compared to other fraction ointments, as shown in Table 6.

#### 3.3.2. Incision Wound Model

The mean tensile strength of group treated by SO increased by 8.36%, which is not statically significant compared to the untreated group. The tensile strength of groups treated with 5% (w/w), 10% (w/w) CEO, and NF significantly (*P* < 0.001) increased by 52.1%, 58.34%, and 57.99%, respectively, when compared to the NC shown in Table 7. There was no significant difference among groups treated with 5% (w/w) CEO, 10% (w/w) CEO, and NF. The groups treated by 10% (w/w) CEO and NF showed higher percentage of tensile strength compared to 5% CEO but failed to reach statistical significance. The 10% (w/w) CEO and the NF had comparable tensile strength, as shown in Table 7.

### 3.4. Evaluation of Anti-Inflammatory

**Figure 5:**
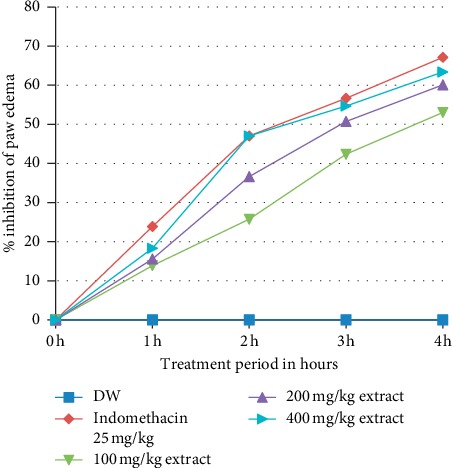
Percent inhibition of oral administered *Hagenia abyssinica* flower crude extract solution on carrageenan-induced paw edema in mice.

The 400 mg/kg dose of crude extract and standard drug showed significant (*P* < 0.05) reduction of paw edema after 1 hour administration of carrageenan injection compared to the negative control, as shown in Table 8. The 100 mg/kg and 200 mg/kg did not show significant difference compared to the negative control after 1 hour administration of carrageenan. After 2 hours of administration of carrageenan injection, 100 mg/kg and 200 mg/kg crude extract showed significant (*P* < 0.01 and *P* < 0.001, respectively) reduction of paw edema in comparison to the negative control. There was significant (*P* < 0.05) difference between 100 mg/kg and 400 mg/kg dose crude extract after 2 hours of administration of carrageenan injection, as shown in Table 8. Maximum percentage of inhibition of edema for the crude extract observed after 4-hour injection of carrageenan by 100, 200, and 400 mg/kg oral dose of crude extract with their respective value 53.05%, 60.09%, and 63.38%. The standard drug showed highest percentage of inhibition with the value of 67.14% after 4 hours of carrageenan administration (Figure 5).

## 4. Discussion

Medicinal plants have been used since time immemorial for treatment of various skin disorders like wound. Medicinal plant favors the rate of wound closure with minimum discomfort, pain, and scarring [37]. Traditionally, the flower of *H. abyssinica* is used for healing of injured skin and wound by applying the powder of flower with honey directly to the injured skin or wound [8, 9]. Applying the plant material and extracts directly to the wound cannot bring the desired activity of the plant. Ointment is must to achieve a sustained drug release at the application sites. The ointment base has role for formation of barrier for moisture over the wound area by hard and white soft paraffin [38–40].

The result of phytochemical screening showed the presence of saponins, tannins, flavonoids, terpenoids, steroids, anthraquinone, and phenolic compounds, which was in line with the previous study [15]. The medicinal values of the plant depend on these phytochemical compounds which produce a definite and specific action on the human body. Phenolic compounds serve in plant defense mechanism to counteract reactive oxygen species [15]. Therefore, the phytochemical screening result reveals that the presence of these phytochemical constituents supports the use of *H. abyssinica* (Bruce) J.F. Gmel in folklore medications.

Both the 5% (w/w) and 10% (w/w) ointment prepared from the crude methanolic extract showed fast wound contraction and reduced epithelization period. This enhanced wound contraction by the CEO might be related to the ability of plant extracts to facilitate proliferation of epithelial cells [41].

In the excision wound model, the crude extract of the flowers of *H. abyssinica* showed statistically significant wound area contraction compared to the NC. The 10% (w/w) CEO showed faster wound area contraction than 5% (w/w) CEO from day 4 onwards. The higher wound contraction rate of the 10% (w/w) CEO was possibly due to either its higher dose of antibacterial effect or induction of macrophage cell proliferation [42].

Previous study of methanolic and dichloromethane flower extract of *H. abyssinica* (Bruce) J.F. Gmel showed antibacterial activity against the pathogens such as *S. aureus*, *P. aeruginosa, E. coli,* and *B. subtilis* which commonly infect wound [15, 43]. This antibacterial activity may contribute to the wound healing effect by keeping the wound free of infections and complications. Such types of agents contribute to the rapid healing of wound [29, 42].

The period of epithelialization was reduced from 18.67 days (simple ointment) to 14.67, 12.67, and 13 days for 5% CEO-, 10% CEO-, and NF-treated groups, respectively. This was due to the ability of *H. abyssinica crude* flower extract to facilitate collagen synthesis, induction of cell proliferation, and antimicrobial activities of bioactive constituents. The CEO reduced the period of epithelialization possibly due to rapid wound contraction that shortens the distance for migrating keratinocytes [44]. The shorter period of epithelialization in the groups treated with the CEO might be related with the antibacterial activity of the flower extracts. The antibacterial activity of the crude extract reduces the exotoxin and endotoxin loads in the wound area which results in reduction of period of epithelization. The ointments might reduce bacteria and endotoxins which allow fibroblasts and epithelial cells to proliferate [45]. Reduction of the period of epithelialization by the extract might be attributed to its ability to enhance contractile property of myofibroblasts and proliferation of epithelial cells around the wound [42]. This is due to the accumulation of fibroblasts, few inflammatory cells further evidenced by skin biopsy of the excision wound at the 12 day showing few concentrations of inflammatory cells as well as more collagen fiber, and proliferating blood capillaries (angiogenesis) compared with control-treated group.

The fraction ointment had different wound healing activities in the excision wound model. The wound closure rate of group treated by 10% (w/w) AQFO was higher than treated by the standard drug on days 6^th^, 8^th^, and 10^th^. This might be due to the presence of more than one bioactive phytochemical constituent that enhances the wound contraction rate [15]. Group treated with 10% (w/w) EAFO had shortest period of epithelization (14 days) and highest rate of contraction of wound compared to other fraction ointment. This may be related to the accumulations of semipolar compound in ethylacetate fraction that promotes the collagen synthesis, migration of myofibroblast, and proliferation of epithelial tissue and anti-inflammatory activity [15, 46]. The higher wound contraction rate of each of the 10% (w/w) fraction ointment may be due to either its higher anti-inflammatory effect or induction of macrophage cell proliferation than each of the 5% (w/w) fractions ointment. The lowest wound healing activity of the CHFO might be due to the presence of lower concentration of the secondary metabolites to achieve wound healing activity [47, 48].

The force required to open the healing wound is known as tensile strength. It also indicates how much the repaired tissue resists to breaking under tension and may indicate in part the quality of repaired tissue [41]. Groups treated by 10% (w/w) and 5% (w/w) CEO and NF required a force which was higher than simple ointment base-treated groups to open the wound on the 10^th^ day. The tensile strength of wounds treated with 10% (w/w) CEO was the highest, but the difference between 10% (w/w) CEO and those treated with NF and 5% (w/w) CEO formulation was not statistically significant. In the incision wound model, the increase in tensile strength of groups treated by 5% (w/w) and 10% (w/w) CEO was due to the remodeling of collagen and the formation of stable intra- and intermolecular crosslink. The collagen molecules synthesized at the wound site and become cross-linked to form fibers. In addition to collagen deposition, matrix deposition and cell migration may contribute to tensile strength of groups treated by the extract ointment [14, 36]. This was evidenced by histological examination which showed the accumulation collagen in CEO-treated groups.

The experimental plant revealed anti-inflammatory activity in the carrageenan-induced paw edema model. This effect might facilitate the wound healing activity of the extract. The anti-inflammatory activity of the plant extract reported here seems to be controversial to the wound healing process, especially in the inflammatory phases. Long duration in the inflammatory phase causes a delay in the healing process. In order to shorten the healing period as well as for minimal pain and scar, anti-inflammatory activity is required [35, 47]. Histopathological examination of the excision wound in the 12 day showed less polymorphonuclear cells and inflammatory cells in groups treated by CEO compared to NC (simple ointment), which evidenced that the plant has anti-inflammatory activity.

Phytochemical analysis of 80% methanolic extract of *H. abyssinica* flowers revealed that tannins, flavonoids, saponins, phenols, terpenoids, and cardiac glycosides are the major constituent of the experimental plants [49]. Saponins and flavonoids have been reported to possess wound healing activity. Terpenoids promote the wound healing process, due to their astringent and antimicrobial activities which seem to be responsible for wound contraction and increased rate of epithelialization [12, 49]. Flavonoids and their derivatives are known to decrease lipid peroxidation by improving vascularity, leading to slowing down of cell necrosis. Polyphones and flavonoids have anti-inflammatory activity by preventing the synthesis of prostaglandins and have antibacterial activity [50]. Glycosides possess antioxidant, antimicrobial, and anti-inflammatory effects. Tannins enhance wound healing by improving regeneration and organization of the new tissue through their astringent and antioxidant properties. Therefore, the presence of this phytochemical constituent in the crude extract may contribute to the wound healing activity [14, 51].

The flame Atomic Absorption Spectrophotometer screening of essential trace metals from the flowers of *H. abyssinica* done previously showed the presence of zinc [52]. There was a possibility the CEO containing zinc. Zinc provides resistance to epithelial apoptosis via cytoprotection, probably through antioxidant activity of the cysteine-rich metallothioneins, against reactive oxygen species and bacterial toxins. Studies have shown that topical administration of zinc is superior to oral administration because of its effect in reducing super infections and necrosis via enhanced local defense systems and the sustained release of zinc ions, which stimulates reepithelialization of wounds. The presence of zinc on the plant extract ointment may improve the moisture holding capacity of skin, complexion, cell migration, and cell regeneration and thus speeds up the wound healing process [53].

Carrageenan-induced paw edema model has been used widely for the evaluation of anti-inflammatory activity of plant extracts. There are three different phases that appeared after carrageenan injection: the first phase (0–1.5 hours) involves the release of histamine and serotonin; the second phase (1.5–2.5 hours) the release of bradykinin and followed by the third phase (2.5–5 hours) involving the production of large amount of proinflammatory mediators such as prostaglandins (PGE2) and proinflammatory cytokines such as interleukin-1 beta (IL-1*β*), interleukin-6 (IL-6), and tumor necrosis factor alpha (TNF*α*); infiltration of neutrophils into the inflammatory site takes place [35, 54].

One-hour after carrageenan injection, only 400 mg/kg dose of crude extract and indomethacin showed significant (*P* < 0.05) inhibition of paw edema compared to the negative control. This may be because the lower doses (100 mg/kg and 200 mg/kg) of the crude flower extract (CFE) might not be able to achieve maximum plasma concentration at 1 h for first phase edema inhibition. After 2 hours administration of carrageenan, the 100 mg/kg and 200 mg/kg of the CFE showed significant inhibition of paw edema. This could be because the lower dose was more effective to inhibit the release of bradykinin and prostaglandin [34]. Maximum percentage of inhibition of edema for the CFE was observed after 4-hour injection of carrageenan by 100, 200, and 400 mg/kg oral dose of CFE with their respective value 53.05%, 60.09%, and 63.38%. This is possible to say that the flowers of *H. abyssinica* contain phytochemical constituents potent and effective in inhibiting the release or the activity of bradykinin and prostaglandins in the third phase of edema formation. The anti-inflammatory activity of the CFE might be associated with secondary metabolites. Flavonoids can significantly inhibit a number of inflammatory mediators and prevent the synthesis of prostaglandins. Terpenoids inhibit phospholipase A2 and block the metabolism of arachidonic acid [41, 55].

## 5. Conclusion

The crude extract exhibited wound healing and anti-inflammatory activities in mice. The wound healing activity elicited by the aqueous and ethyl acetate fraction corroborates with the folkloric practice that the healers infused the flowers of *H. abyssinica* (Bruce) J.F. Gmel with water for topical application. The results of this study support the medicinal use of flowers of *H. abyssinica* (Bruce) J.F. Gmel for wound healing.

## Figures and Tables

**Table 1:** Formula use for preparation of simple and medicated ointment.

Ingredient	MF (g)	RF (g)
Wool fat	50	10
Hard paraffin	50	10
White soft paraffin	850	170
Cetostearyl alcohol	50	10
Total	1000	200

MF: master formula; RF: reduced formula.

**Table 2:** Phytochemical constituent of methanolic extract of flower of *H. abyssinica*.

Secondary metabolite	Test results
Alkaloid	−
Flavonoids	+
Saponins	+
Tannins	+
Glycosides	+
Phenols	+
Steroids	+
Terpenoids	+
Anthraquinones	+

−, absence; +, present.

**Table 3:** Activity of topical application of the 80% methanolic extract of the flower of *H. abyssinica* on the wound area of the excision wound model in mice.

Wound area (mm^2^) (mean ± SEM) postwounding days				
Day	SO	NF	5% CEO	10% CEO
0	311.13 ± 1.30	311.33 ± 1.38	312.50 ± 0.85	311.33 ± 1.31
2	303.13 ± 4.19	292.71 ± 1.92	297.92 ± 3.09	292.71 ± 1.92
4	273.96 ± 2.98	195.83 ± 4.47^a3^	251.04 ± 4.68^a2^	194.79 ± 1.92^a3^
6	179.17 ± 3.09	123.96 ± 7.11^a3^	148.96 ± 4.39^a2^	117.71 ± 3.76^a3^
8	142.71 ± 2.98	55.21 ± 2.98^a3^	78.13 ± 2.68^a3^	53.13 ± 2.68^a3^
10	94.79 ± 2.98	18.75 ± 1.61^a3^	38.54 ± 2.51^a3^	18.75 ± 2.8^a3^
12	64.58 ± 5.02	7.29 ± 3.76^a3^	19.79 ± 1.92^a3^	4.17 ± 3.09^a3^
14	38.54 ± 4.39	0	4.17 ± 2.64^a3^	0
16	23.96 ± 4.95	0	0	0

*Note.* Values are expressed as mean ± SEM (*n* = 6 mice in each group) and analyzed by one-way ANOVA followed by the post hoc Tukey test; ^a^compared to the negative control; ^b^compared to the 5% CEO; ^2^*P* < 0.01; ^3^*P* < 0.001; SO = simple ointment; NF = nitrofurazone; CEO = crude flower extract ointment.

**Table 4:** Activity of topical application of the solvent fraction extract ointment of the flower of Hagenia *abyssinica* on wound contraction of excision wound model in mice.

Wound area (mm^2^) (mean ± SEM) (% contraction)								
Day	SO	NF	5% AQFO	10% AQFO	5% CHFO	10% CHFO	5% EAFO	10% EAFO
0	312.67 ± 1.09	311.33 ± 0.71	312.25 ± 0.95	312.67 ± 0.76	312.67 ± 0.56	311.83 ± 0.87	312.83 ± 0.79	312.00 ± 0.77
2	304.17 ± 3.49 (2.72%)	288.54 ± 2.96 (7.29%)^a1^	296.88 ± 2.68 (4.92%)	288.54 ± 2.98 (8.72%)^a1^	300 ± 2.28 (4.05%)	296.88 ± 3.52 (4.79)	293.75 ± 3.23 (6.10%)	286.46 ± 2.98 (8.19%)^a2^
4	286.46 ± 3.49 (8.38%)	251.04 ± 2.98 (19.37%)^a2^	271.86 ± 2.77 (12.94%)^a1d3^	259.36 ± 3.52 (17.05%)^a2^	276.04 ± 2.51 (11.72%)^b3c2d3e1^	270.83 ± 3.09 (13.15)^a1b2d3^	261.46 ± 2.98 (16.42%)^a2d1^	247.92 ± 3.09 (20.54%)^a3^
6	201.04 ± 2.98 (35.70%)	173.96 ± 3.78 (44.12%)^a3^	184.38 ± 3.52 (40.95%)^a1d3^	170.83 ± 2.08 (45.36%)^a3^	189.58 ± 4.17 (39.37%)^b1c2d3^	185.42 ± 2.64 (40.54)^a1c1d3^	186.46 ± 2.98 (40.40%)^a1c1d3^	162.50 ± 2.28 (47.92%)^a3^
8	98.96 ± 1.92 (68.35%)	75.00 ± 3.76 (75.91%)^a3^	81.25 ± 2.28 (73.98%)^a2c1d3^	67.71 ± 2.98 (78.34%)^a3^	81.25 ± 2.28 (74.01%)^a1c1d3^	80.21 ± 2.98 (74.28)^a1d3^	78.13 ± 2.68 (75.02%)^a2d3^	55.21 ± 2.98 (82.30%)^a3b3^
10	71.88 ± 2.68 (77.01%)	35.42 ± 3.09 (88.62%)^a3^	51.04 ± 2.28 (83.65%)^a2b1c2d3^	31.25 ± 2.28 (90.01%)^a3^	55.21 ± 1.92 (82.34%)^a1b2c3d3e1^	39.58 ± 2.08 (87.31)^a3d2^	41.69 ± 4.47 (86.67%)^a3d3^	19.79 ± 3.76 (93.66%)^a3b1^
12	43.75 ± 2.68 (86.01%)	10.42 ± 3.49 (96.65%)^a3^	26.04 ± 2.98 (91.66%)^a2b2d2^	16.67 ± 2.64 (94.67%)^a3^	30.21 ± 3.76 (90.34%)^a1b3c1d3^	22.92 ± 2.08 (92.65)^a3d2^	20.33 ± 1.94 (93.50%)^a3d1^	9.37 ± 2.68 (97.66%)^a3^
14	25.00 ± 2.28 (92.00%)	3.13 ± 2.13 (100%)	10.38 ± 3.48 (96.68%)^a2^	2.08 ± 1.32 (99.33%)^a3^	13.54 ± 2.98 (95.67%)^a1b2c1d2^	9.38 ± 3.13 (97.00)^a2^	7.33 ± 1.92 (97.66%)^a3^	1.04 ± 1.04 (99.67%)^a3^
16	13.54 ± 2.98 (95.67%)	0 (100%)	3.13 ± 2.13 (99.00%)^a2^	0 (100%)^a3^	3.13 ± 2.13 (99.00%)^a2^	1.08 ± 1.04 (99.65)^a3^	0 (100%)^a3^	0 (100%)^a3^

*Note.* Values are expressed as mean ± SEM (*n* = 6 mice in each group) and analyzed by one-way ANOVA followed by the post hoc Tukey test; ^a^compared to the negative control; ^b^compared to positive control; ^c^compared to 10% AQFO; ^d^compared to 10% EAFO; ^e^compared to 5% EAFO; ^1^*P* < 0.05; ^2^*P* < 0.01; ^3^*P* < 0.001; AQFO = aqueous fraction ointment; CHFO = chloroform fraction ointment; EAFO = ethylacetate fraction ointment; NF = nitrofurazone 0.2% ointment; SO = simple ointment.

**Table 5:** Activity of the 80% methanolic crude extracts ointment of the flowers of *H. abyssinica* on period of epithelialization (number of days).

Treatment group	Period of epithelization (days), mean ± SEM	% decrease in epithelization period
SO	18.67 ± 0.67	—
NF	13.00 ± 0.45^a3^	30.37%
5% CFEO	14.67 ± 0.42^a3^	21.42%
10% CFEO	12.67 ± 0.42^a3^	32.14%

Values are expressed as mean ± SEM (*n* = 6 mice in each group) and analyzed by one-way ANOVA followed by the post hoc Tukey test; ^a^compared to the negative control; ^3^*P* < 0.001; SO = simple ointment; NF = nitrofurazone 0.2% ointment; CEO = crude flower extract.

**Table 6:** Activity of the solvent fraction extract ointment of the flowers of *H. abyssinica* on period of epithelialization (number of days).

Treatment group	Period of epithelization (day), mean ± SEM	% decrease in epithelization period
SO	18.33 ± 0.61	—
NF	14.67 ± 0.42^a2^	20.00
5% AQFO	15.67 ± 0.613^a1^	14.51
10% AQFO	14.67 ± 0.42^a2^	20.00
5% CHF	16.33 ± 0.61	10.91
10% CHFO	15.67 ± 0.61^a1^	14.51
5% EAFO	15.33 ± 0.42^a2^	16.37
10% EAFO	14.00 ± 0.52^a3^	23.62

Values are expressed as mean ± SEM (*n* = 6 mice in each group) and analyzed by one-way ANOVA followed by the post hoc Tukey test; ^a^compared to the SO; ^1^*P* < 0.05; ^2^*P* < 0.01; ^3^*P* < 0.001; AQFO = aqueous fraction ointment; CHFO = chloroform fraction ointment; EAFO = ethylacetate fraction ointment; NF = nitrofurazone 0.2% ointment; SO = simple ointment.

**Table 7:** The effects of 80% methanolic crude extract ointment of Hagenia *abyssinica* flowers on tensile strength of incision wound model in mice.

Treatment group	Tensile strength (g) (mean ± SEM)	% of tensile strength
LU	209.76 ± 1.73	—
SO	227.29 ± 4.64	8.36
NF	359.10 ± 4.44^a3b3^	57.99
5% (w/w) CEO	346.65 ± 3.62^a3b3^	52.51
10% (w/w) CEO	359.89 ± 7.12^a3b3^	58.34

*Note.* Values are expressed as mean ± SEM (*n* = 6 mice in each group) and analyzed by one-way ANOVA followed by the post hoc Tukey test; LU = left untreated; ^a^compared to LF; ^b^compared to SO-treated group; ^3^*P* < 0.001.

**Table 8:** Anti-inflammatory activity of the crude extract on carrageenan-induced paw edema.

Treatment group	The paw volume (ml), mean ± SEM				
	Basal	1 h	2 h	3 h	4 h
Control	0.182 ± 0.01	0.180 ± 0.008	0.202 ± 0.014	0.203 ± 0.01	0.213 ± 0.008
Indomethacin	0.167 ± 0.008	0.137 ± 0.004^a2^	0.107 ± 0.003^a3^	0.088 ± 0.007^a3^	0.070 ± 0.004^a3^
100 mg/kg extract	0.195 ± 0.008	0.155 ± 0.008	0.150 ± 0.004^a2b2c2^	0.117 ± 0.005^a3b1^	0.100 ± 0.004^a3b2c1^
200 mg/kg extract	0.193 ± 0.012	0.152 ± 0.008	0.128 ± 0.008^a3^	0.100 ± 0.004^a3^	0.085 ± 0.002^a3^
400 mg/kg extract	0.178 ± 0.012	0.147 ± 0.005^a1^	0.107 ± 0.004^a3^	0.092 ± 0.005^a3^	0.078 ± 0.005^a3^

*Note.* Values are expressed as mean ± SEM (*n* = 6 mice in each group) and analyzed by one-way ANOVA followed by the post hoc Tukey test; ^a^compared to the negative control; ^b^compared to indomethacin; ^c^compared to 400 mg/kg; ^1^*P* < 0.05; ^2^*P* < 0.01; ^3^*P* < 0.001.

## Data Availability

The original data used to support the findings of this study are available from the corresponding author and institutional review board of University of Gondar upon request.
